# Olfaction in *Parkin* carriers in Chinese patients with Parkinson disease

**DOI:** 10.1002/brb3.680

**Published:** 2017-03-28

**Authors:** Ying Wang, Jian‐Jun Wu, Feng‐Tao Liu, Kui Chen, Chen Chen, Su‐Shan Luo, Yi‐Xuan Wang, Da‐ke Li, Rong‐Yuan Guan, Yu‐Jie Yang, Yu An, Jian Wang, Yi‐Min Sun

**Affiliations:** ^1^ Department and Institute of NeurologyHuashan HospitalFudan UniversityShanghaiChina; ^2^ Department of NeurologyJing'an District Center Hospital of ShanghaiShanghaiChina; ^3^ Institute of Biomedical SciencesMedical SchoolFudan UniversityShanghaiChina

**Keywords:** gene, olfaction, *Parkin*, Parkinson disease, Sniffin’ Sticks test

## Abstract

**Background:**

Olfactory identification was reported to be better among PD (Parkinson disease) patients with *Parkin* mutations, but previous studies didn't eliminate the interference of other PD related genes on olfaction, and whether olfaction of *Parkin* mutations patients was better in Chinese population was still unknown.

**Objective:**

To assess olfaction function among PD patients with *Parkin* mutations in Chinese population.

**Materials and Methods:**

A total of 226 PD patients with a positive family history or an early‐onset age (<50 years) were enrolled for genetic testing of PD related genes by target sequencing and multiple ligation‐dependent probe amplification. The clinical data including olfactory function test were investigated. Linear regression was performed to adjust for the covariates between all groups.

**Results:**

There were 68 patients found having a negative result in PD genetic testing and 43 patients carrying homozygous or compound heterozygous *Parkin* mutations. Among them, 49 PD panel negative patients and 33 PD‐*Parkin* patients had results of olfactory assessment. PD *‐Parkin* patients performed significantly better on the Sniffin’ Sticks tests than panel negative patients (8.0 ± 1.7 vs. 5.7 ± 1.9, *p *<* *.001), but still worse compared to healthy controls (9.4 ± 1.5, *p *=* *.003). These differences persisted after adjusting for confounders.

**Conclusions:**

Among Chinese population, PD *‐Parkin* patients had relatively preserved olfaction compared to PD panel negative patients after eliminating the interference of other PD related genes, but were still worse than healthy controls.

## 1 Introduction

Olfactory impairment is one of the earliest manifestations of idiopathic Parkinson disease (PD), which could predate a clinical diagnosis by at least 4 years (Ross et al., 2008). Olfaction test has been proposed as a useful tool for screening those who have a higher risk for development of PD later (Chaudhuri, Healy, & Schapira, 2006; Ross et al., 2008). Neuropathological advances suggested that the olfactory system was among the earliest brain regions involved in PD (Del Tredici, Rub, De Vos, Bohl, & Braak, 2002) and olfactory deficits were associated with the presence of incidental Lewy bodies in the brains of decedents without Parkinsonism or dementia during life (Ross et al., 2006).

Parkinsonism associated with *Parkin* gene mutation was one of the most common familial forms of PD, which was characterized by early onset of symptoms (mainly before age 50), slow progression, elective dopaminergic neuronal loss and the absence of Lewy bodies (Gouider‐Khouja et al., 2003; Lucking et al., 2000; Mizuno, Hattori, & Matsumine, 1998). Based on the special clinical and pathological features, *Parkin* mutation causing Parkinsonism has been postulated as a different disease entity compared to idiopathic PD (Doherty et al., 2013; Khan et al., 2004). While impaired olfaction was frequently associated with PD, olfactory identification was reported to be better among patients with *Parkin* mutations (Alcalay et al., 2011; Malek et al., 2016). This was in line with pathologic findings in PD patients that *Parkin* mutation carriers showed less extensive distribution of Lewy bodies, which might not follow Braak staging and spares olfactory structures (Doherty et al., 2013; Gouider‐Khouja et al., 2003; Hayashi et al., 2000).

Khan and colleagues first assessed whether olfactory function differed in *Parkin* related PD patients compared with early onset PD patients (EOPD) in 2004, and found that olfaction scores of *Parkin* group were better than EOPD group (Khan et al., 2004). Alcalay and colleagues further compared olfaction between *Parkin* single heterozygotes and compound heterozygotes. Among PD patients, compound heterozygotes had higher University of Pennsylvania Smell Identification Test scores (UPSIT) than heterozygotes or noncarriers (Alcalay et al., 2011). Until now, whether *Parkin* mutation carriers in Chinese population had the same olfaction manifestation as Caucasian populations was still unknown. Previous studies about olfaction function in patients of PD with *Parkin* mutations only excluded several special genes’ interference (Alcalay et al., 2011; Khan et al., 2004; Malek et al., 2016). Whether the patients carried other PD related genes that might influence olfaction was not clear. In addition, their sample size was relatively small. In order to assess olfaction function in *Parkin* related PD patients among Chinese population, we evaluated olfactory function in a cohort of Chinese PD patients with *Parkin* mutations by a gene panel containing all known PD related genes.

## 2 Methods

### 2.1 Subject

New and follow‐up PD patients with a positive family history or an early‐onset age (<50 years), were enrolled consecutively from the Movement Disorders Clinic at Huashan Hospital from June 1, 2014 to April 30, 2016 for genetic testing. The clinical materials of these patients were investigated retrospectively especially for the olfactory assessment. Thirty‐four gender matched subjects were voluntarily recruited from the community as controls, who had no neurological disorders, psychiatric disorders or sinonasal diseases.

The diagnosis of PD was according to the United Kingdom Parkinson's Disease Society Brain Bank criteria for idiopathic PD (Daniel & Lees, 1993). The written informed consent was obtained from each subject after the aims and protocol was fully explained. The project was approved by the Huashan Hospital Institutional Review Board.

### 2.2 Genetic testing and variant analysis

A panel of 40 PD related genes including *Parkin* was designed (Table S1). The genetic analysis was carried out by target sequencing and multiple ligation‐dependent probe amplification (MLPA) as previously reported (Huang et al., 2013). Briefly, all exons and their corresponding flanking regions of the genes in the panel were selected as target regions. DNA (Deoxyribonucleic Acid) from the peripheral blood of the patients was prepared as an Illumina sequencing library which were enriched for these target regions. The captured libraries were then sequenced using IlluminaNextSeq500 Sequencer with a sequencing depth of 200 × . The raw data was then compared to the reference sequence provided by Mygenostics Inc by standardized procedures (Huang et al., 2013).

The variants on all suspected variants were further confirmed by sanger sequencing using standardized procedure.

The MLPA was carried out by the kit of SALSA MLPA probemix P051‐D1/P052‐D2 Parkinson (MRC‐Holland) according to the protocol provided by the manufacture.

### 2.3 Variant analysis

The variants on the exonic and splicing sites (within 100 bp of a splice junction) were analyzed. Mutation reads <5 and mutation frequency <30% were filtered out. Synonymous variant and the variants whose allele frequencies were higher than 1% in 1000Genome, ESP6500 and inhouse databases were excluded. HGMD professional (RRID:SCR_001888) and Clinvar (http://www.ncbi.nlm.nih.gov/clinvar) database (RRID:SCR_006169) were used to detect the pathogenic level of the variants.

The variants not included in these two databases were further classified by the American College of Medical Genetics and Genomics and the Association for Molecular Pathology (ACMG) Standards using the terms of ‘pathogenic’, ‘likely pathogenic’, ‘uncertain significance (VUS)’, ‘likely benign’ and ‘benign’ (Richards et al., 2015).

### 2.4 Clinical assessments

All evaluations and tests were carried out in those PD patients after at least 12 hr withdrawal of dopaminergic medication. A detailed assessment of clinical materials, neurologic examination including Hoehn and Yahr scale, Unified Parkinson disease rating scale (UPDRS) motor examination were done as previously reported (Xiong et al., 2016).

### 2.5 Olfactory assessment

Olfactory function test was performed with a 12‐item Sniffin’ Sticks tests, according to the manufacturer's instructions (Hummel, Kobal, Gudziol, & Mackay‐Sim, 2007). The test was conducted on bilateral nostrils. Each stick was held approximately 2 cm in front of the nostrils for 2–3 s, with an interval of 20–30 s between each stick. The patient identified a smell by selecting one out of four possible answers from multiple choice standard cards as previously reported (Huang et al., 2016). Test scores ranged from 0 to 12 and higher scores indicate better olfaction. All examiners were unaware of the genetic status of the participants at the time of recruitment and thereafter.

Olfactory examination was conducted at least 1 hr after the last cigarette, meal or beverage. Patients with sinonasal disorders or a cold were not tested.

### 2.6 Statistical analysis

Demographics and clinical characteristics were compared among the PD‐*Parkin* patients and PD panel negative patients. Student *t* test was used for continuous value, and χ^2^ test for categorical data as appropriate. Age and olfaction performance were compared among the PD‐*Parkin* patients, PD panel negative patients and healthy controls using one way ANOVA, and the post hoc tests were conducted by Bonferroni multiple comparison tests. Multiple forward linear regression analyses were used to adjust for the covariates between all the groups, including current age, education, Mini‐Mental State Examination (MMSE), and levodopa equivalent daily dose (LEDD). *p *<* *.05 was considered significant. All analyses were performed with SPSS version 22.0 statistical software (RRID:SCR_002865).

## 3 Results

### 3.1 Coverage in the target region

The average sequencing depth of the target region was 417.8× and the mean percentage of the target region covered at least 20× or 10× was 92.45% or 96.57% respectively. 625 exons were sequenced with 5 (0.8%) exons having reads depth below 10× (Table S2). These areas were mainly in repetitive or GC rich area.

### 3.2 Sample characteristics

A total of 226 PD patients met the criteria of age at onset <50 or positive family history and enrolled in the current study. According to the genetic testing, 68 patients had no mutations or variants ranked as pathogenic, likely pathogenic or VUS detected by the panel. 43 patients carried homozygous and compound heterozygous *Parkin* mutations only. The other 115 patients were with 1) other causative PD mutations or 2) risk variants (including two patients with both *Parkin* single heterozygous mutation and other PD‐related risk variants, six patients with single heterozygous mutation) or 3) variants ranked as likely pathogenic or VUS or 4) homozygous *Parkin* mutations plus one other PD‐related risk variant (one patient).

**Figure 1:**
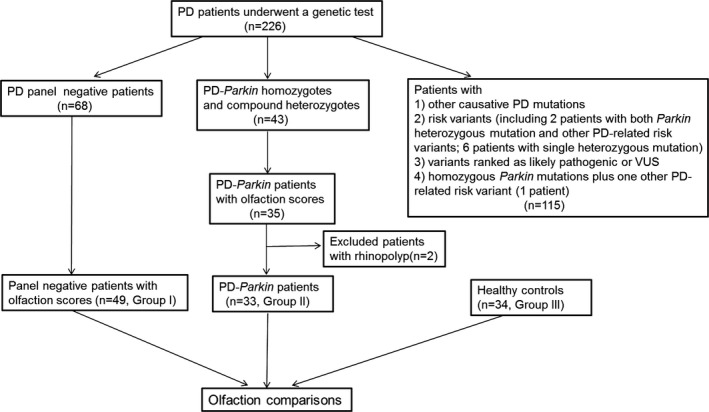
Enrollment scheme of the study

Among all the PD panel negative and PD‐*Parkin* patients, 49 (group I) and 35 had results of olfactory assessment. Two PD‐*Parkin* patients were excluded for the history of rhinopolyp. The rest 33 were assigned to group II. And the 34 controls from community were included in group III. The detailed procedure was shown in Figure 1.

The clinical details of patients in group I, group II and group III were shown in Table 1. Age at onset was significantly earlier and disease duration was significantly longer in group II. There were no statistic difference in UPDRS III (*p *=* *.818), Hoehn and Yahr stage (*p *=* *.193), LEDD (*p *=* *.100), gender (*p *=* *.898), family history (*p *=* *.342), MMSE (*p *=* *.952) and education (*p *=* *.631) between group I and group II.

**Table 1:** Demographics, disease characteristics and olfaction performance of PD panel negative group, PD‐*Parkin* group and healthy controls

	PD panel negative group (Group I) (*n *= 49)	PD‐*Parkin* group (Group II) (*n *= 33)	Healthy controls (Group III) (*n *= 34)	*p*‐value (Three groups)	*p*‐value (Group I vs. Group II)	*p*‐value (Group II vs. Group III)	*p*‐value (Group I vs. Group III)
Olfaction score	5.7 ± 1.9 (2–9)	8.0 ± 1.7 (5–11)	9.4 ± 1.5 (6–12)	<.001^a^	<.001^b^	.003^b^	<.001^b^
Current age (years)	50.3 ± 10.9 (17–73)	35.1 ± 7.4 (24–49)	62.7 ± 3.7 (57–73)	<.001^a^	<.001^b^	<.001^b^	<.001^b^
Age at onset	45.4 ± 10.7 (13–66)	25.1 ± 5.7 (14–41)	–	–	<.001	–	–
Disease duration (months)	60.7 ± 40.5 (9–193)	119.1 ± 71.3 (19–280)	–	–	<.001	–	–
Male sex (% male)	35 (71)	24 (73)	24 (71)	.981	.898	.846	.934
Family history (%)	23 (47)	12 (36)	–	–	.342	–	–
Education (years)	11.6 ± 3.4 (5–19)	12.0.±3.3 (6–17)	–	–	.631	–	–
Hoehn & Yahr stage	2.2 ± 0.7 (1–4)	2.4 ± 0.6 (1–4)	–	–	.193	–	–
UPDRS–III (“off” medication)	34.2 ± 13.2 (6–64)	34.9 ± 12.6 (14–68)	–	–	.818	–	–
LEDD (mg/day)	479.3 ± 400.6 (0–2251.2)	348.4 ± 252.6 (0–1100)	–	––	.100	–	–
MMSE score	27.9 ± 2.3 (19–30)	28.0 ± 2.0 (23–30)	–	–	.952	–	–

UPDRS, Unified Parkinson's Disease Rating Scale; LEDD, levodopa equivalent daily dose; PD, Parkinson disease.

Data were shown as mean ± *SD* (range) except where indicated. All statistical analyses were done by *t* tests for continuous variables and by χ^2^ test for categorical variables as appropriate unless stated otherwise ^a^One way ANOVA. ^b^Post hoc tests were conducted by Bonferroni multiple comparison test.

### 3.3 Olfaction assessment in PD patients and controls

**Figure 2:**
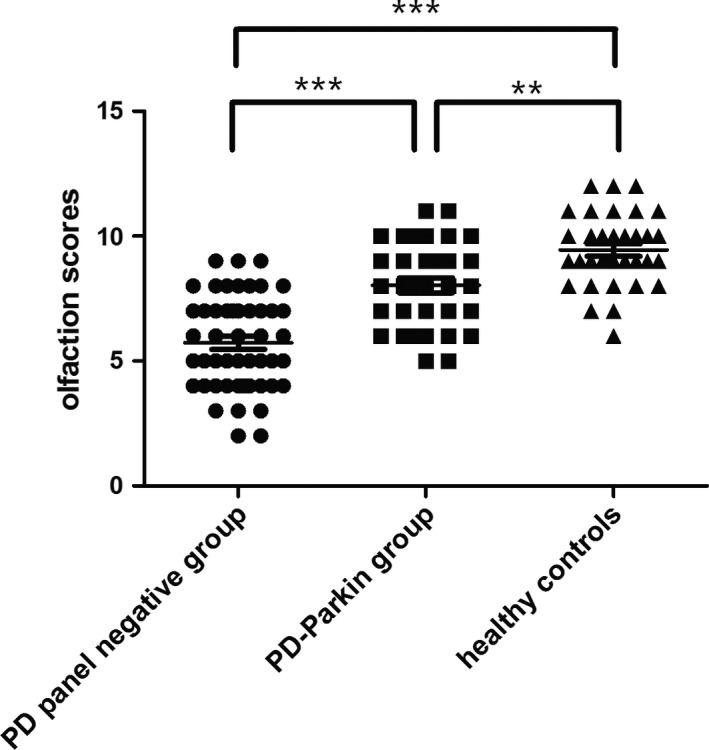
Scatter diagram of olfaction scores in Parkinson disease (PD) panel negative group, PD‐*Parkin* group and healthy controls. **p < .01, ***p < .001

Group II performed significantly better (8.0 ± 1.7) than group I (5.7 ± 1.9) on the Sniffin’ Sticks test scores (*p *<* *.001), despite longer disease duration (Figure 2 and Table 1). In a linear regression model including all PD patients, the PD‐*Parkin* patients were associated with higher olfaction scores when compared with the PD panel negative patients (*p = *.005), after adjustment for age, education, MMSE and LEDD. In addition, the olfaction test scores were still significantly lower in the PD‐*Parkin* patients compared with healthy controls (*p = *.003), and the differences persisted after adjustment for age (*p *=* *.001) (Figure 2 and Table 1).

Patients in group II were further categorized by compound heterozygotes and homozygotes, but we didn't find any significant difference in olfaction scores between them (*p = *.222). Though statistical significance was not found between the *Parkin* single heterozygotes and *Parkin* homozygotes/compound heterozygotes due to the small number of heterozygotes (*n *= 6), the mean olfaction score of heterozygotes was lower than that of the patients in group II (5.3 vs. 8.0).

## 4 Discussion

*Parkin* related PD had clinical and pathologic features that might show varying degrees of difference with idiopathic PD (Doherty et al., 2013). Young onset age, more symmetric involvement, dystonia at presentation, brisk deep tendon reflexes, a good response to levodopa therapy (Lohmann et al., 2003; Lucking et al., 2000), and more symmetric and more marked reduction of dopamine uptake on 123I FP‐CIT SPECT scan (DaTSCAN) were found in *Parkin*‐related disease (McNeill et al., 2013). *Parkin* mutation carriers (homozygotes or compound heterozygotes) also showed better cognitive and motor performance than noncarriers, suggesting a slower disease progression (Alcalay et al., 2014). Our study found that the PD*‐Parkin* group had relatively preserved olfaction compared with the PD panel negative group in Chinese PD patients after eliminating the interference of other PD related genes, which further supported the hypothesis that *Parkin*‐related PD was a different clinicopathologic entity to idiopathic PD.

Olfaction had been the subject of few prior studies with detailed *Parkin* mutation analysis in patients with PD (Alcalay et al., 2011; Malek et al., 2016). Assessing olfactory function in *Parkin* related PD was important for a better understanding of the underlying pathophysiology and for the clinical recognition of the *Parkin* phenotype. Previous study found that the mean UPSIT score in *Parkin* patients with early‐onset Parkinsonism was higher than the *Parkin*‐negative group (Khan et al., 2004). Later, a US study showed that olfaction in young onset PD was related to *Parkin* mutation status, and those with compound heterozygous mutations had preserved olfactory function, unlike those with single heterozygous mutations and noncarriers (Alcalay et al., 2011). However, all the previous studies only detected and excluded certain genes. Whether other undetected PD related genes had an influence on the olfaction manifestation was not clear. In our study, we compared olfactory function among patients with PD grouped by the *Parkin* mutation status and found that olfactory function was better in the patients harboring 2 *Parkin* mutations compared to those PD panel negative patients, but still worse than healthy controls. According to our findings, the mean onset age and disease duration was significantly longer in the PD‐*Parkin* group than the PD‐panel negative group. However, olfactory test scores were independent of the disease stage and duration (Doty, Deems, & Stellar, 1988). Thus, the different total olfaction scores between the two groups were not due to the different onset age and disease duration. In our study, the genes were detected by the target sequencing and MPLA, which could identify both sequence and dosage mutations in patients with PD. Our control group of *Parkin* negative patients referred to those with no mutations or variants ranked as pathogenic, likely pathogenic or VUS detected by the panel, thus eliminated the interference of other genes on olfaction (Marras et al., 2011; Saunders‐Pullman et al., 2011). This was the most significant difference from previous studies (Alcalay et al., 2011; Malek et al., 2016). We also had olfaction scores of healthy people as an internal control group. Meanwhile, this was the first study about olfaction in a cohort of Chinese patients with *Parkin*‐linked PD with a large sample size. Thus, our data strongly supported that the preserved olfaction in the PD patients harboring 2 *Parkin* mutations compared to idiopathic PD was one component of a wider pattern of distinguishing features. We didn't find any significant difference in the olfaction scores between the compound heterozygotes and homozygotes (*p = *.222). This was consistent with previous reports that harboring 2 *Parkin* mutations typically lead to juvenile‐onset or early‐onset forms of PD (Abbas et al., 1999; Lucking et al., 2000). Statistical significance was not found between the *Parkin* heterozygotes and *Parkin* homozygotes/compound heterozygotes due to the small number of heterozygotes, but the mean olfaction score of heterozygotes was lower than that of the patients in group II, which was consistent with previous reports that heterozygous *Parkin* mutations is regard as risk factors of PD (Alcalay et al., 2011; Malek et al., 2016).

Limitations of our study included its sample size and heterozygote number, which did not allow us to analyze the olfaction in the single heterozygotes and carriers with 2 *Parkin* mutations, separately. Another limitation of our study was that the clinical materials of the patients were investigated retrospectively. Though the patients were collected continuously, the olfaction data was not, which meant not all the PD patients had an olfactory examination. For the data analysis in our study, we chose the patients with results of both genetic test and olfactory assessment, retrospectively. Prospective studies were needed to confirm our results in the future.

Better performance on the olfaction scores might reflect a different distribution of pathology among the *Parkin* positive patients compared with the negative patients. In PD, degeneration of the olfactory nucleus with impairment of smell starts early in Braak stageI(Braak et al., 2003) and was thought to be specific for Lewy body‐type neurodegeneration. The PD*‐Parkin* patients had less extensive distribution of Lewy bodies neuropathology, that might not follow Braak staging and spares olfactory structures (Doherty et al., 2013; Gouider‐Khouja et al., 2003), and might explain their preserved olfaction. The absence of Lewy bodies in patients with *Parkin* mutations suggested that *Parkin* might be required for the formation of Lewy bodies. Indeed, researchers had found that familial‐linked mutations in *Parkin* disrupted the ubiquitination of synphilin‐1 and the ubiquitination of the Lewy‐body–like inclusions (Chung et al., 2001; Lim et al., 2005). Others hold the opinion that patients with a younger age at onset might have a different mechanism for dealing with abnormal protein accumulation. As a result, only neuronal dropout and gliosis were the neuropathologic findings in *Parkin* patients with autopsy (Doherty & Hardy, 2013)**.** However, in recent years, Lewy bodies pathology, the typical of idiopathic PD, was found at autopsy in a minority of cases with *Parkin* mutations (Farrer et al., 2001; Miyakawa et al., 2013; Pramstaller et al., 2005). It remained unclear how different mutations in the *Parkin* gene resulting in a similar loss of function could lead to different genetic and neuropathologic features. Autopsy data of Lewy bodies in olfactory bulb were lacking, further research was required to address the relationship between olfactory performance and the underlying disease mechanism.

## 5 Conclusion

Our overall finding of better olfaction in PD‐*Parkin* patients in Chinese PD patients was similar with those in other population. Quantitative measures of olfaction might assist in distinguishing *Parkin* carriers from other forms of young‐onset PD and idiopathic PD.

## Conflict of Interest

The authors declare no financial or other conflict of interest.
