# Supplementary material for: Olfaction in Parkin carriers in Chinese patients with Parkinson disease
Source: Brain Behav. 2017 Mar 28;7(5):e00680. doi: 10.1002/brb3.680 (PMC5434185; doi:10.1002/brb3.680)
Supplement: Supplementary file 1 [file BRB3-7-e00680-s001.docx]

**Supplementary Table 1** The Parkinson’s disease related genes tested in the cohort

| *ADCY5* | *ADH1C* | *ANO3* | *ATP1A3* | *ATP6AP2* | *ATP13A2* | *CIZ1* |
| --- | --- | --- | --- | --- | --- | --- |
| *DJ1* | *DNAJC6* | *DRD2* | *EIF4G1* | *FA2H* | *FBXO7* | *GBA* |
| *GCH1* | *GIGYF2* | *GNAL* | *HTRA2* | *LRRK2* | *MAPT* | *PANK2* |
| *PARK2* | *PINK1* | *PLA2G6* | *PRKRA* | *PRRT2* | *RAB39B* | *SGCE* |
| *SLC2A1* | *SNCA* | *SPR* | *SYNJ1* | *TAF1* | *TBP* | *TH* |
| *THAP1* | *TOR1A* | *TUBB4A* | *UCHL1* | *VPS35* |  |  |
