# Supplementary material for: Olfaction in Parkin carriers in Chinese patients with Parkinson disease
Source: Brain Behav. 2017 Mar 28;7(5):e00680. doi: 10.1002/brb3.680 (PMC5434185; doi:10.1002/brb3.680)
Supplement: Supplementary file 2 [file BRB3-7-e00680-s002.doc]

**Supplementary table 2** The exons with reads depth below 10x in the genetic testing

| Gene | Exon number | GC% |
| --- | --- | --- |
| *VPS35* | 1 | 71.5% |
| *FA2H* | 1 | 77.7% |
| *SYNJ1* | 1 | 74.1% |
| *SYNJ1* | 25 | 69.8% |
| *EIF4G1* | 3 | 61.0% |
| *TOR1A* | 1 | 71.2% |
